# Identification of EMT-Related lncRNAs as Potential Prognostic Biomarkers and Therapeutic Targets for Pancreatic Adenocarcinoma

**DOI:** 10.1155/2022/8259951

**Published:** 2022-04-11

**Authors:** Yanyao Deng, Hai Hu, Le Xiao, Ting Cai, Wenzhe Gao, Hongwei Zhu, Shuai Wang, Jixing Liu

**Affiliations:** ^1^Department of Rehabilitation, The First Hospital of Changsha, Changsha, Hunan Province, China; ^2^Department of Orthopedics, The Third Xiangya Hospital, Central South University, Changsha, China; ^3^Department of Gastroenterology, The Third Xiangya Hospital, Central South University, Changsha, China; ^4^Department of Hepatopancreatobiliary Surgery, The Third Xiangya Hospital, Central South University, Changsha, China; ^5^Department of Nephrology, Institute of Nephrology, 2nd Affiliated Hospital of Hainan Medical University, Hainan, China

## Abstract

Epithelial-mesenchymal transition (EMT) can promote carcinoma progression by multiple mechanisms; many studies demonstrated the invasiveness of pancreatic adenocarcinoma (PAAD) associated with the EMT, but how it acts through an lncRNA-dependent manner is unknown. Here, we investigated 146 samples from The Cancer Genome Atlas (TCGA) and 92 samples from the International Cancer Genome Consortium (ICGC). By gene set variation analysis (GSVA) and weighted correlation network analysis (WGCNA), we explored the EMT-related long noncoding RNAs (EMTlnc). Then, we performed univariate Cox regression analysis to screen their prognostic value for PAAD. The least absolute contraction and selection operator (LASSO) Cox regression was used to establish EMT-related lncRNA prognostic signal (EMT-LPS). In addition, we established a competitive endogenous ceRNA network. Then, we identified 33 prognostic EMTlnc as prognostic lncRNAs and established an EMT-LPS which showed strong prognostic ability in stratification analysis. By corresponding risk scores, patients were divided into low-risk and high-risk subgroups. Principal component analysis (PCA) showed that these subgroups had individual EMT status. Enrichment analysis showed that in the high-risk subgroup, biological processes, pathways, and hallmarks related to malignant tumors are more common. What is more, we constructed a nomogram that had powerful ability to predict the overall survival rate (OS) of PAAD patients in two datasets. So, EMT-LPS are a principal element in PAAD's carcinoma progression and may help us in choosing the way of prognosis assessment and provide some clues to design the new drugs for PAAD.

## 1. Introduction

Pancreatic adenocarcinoma (PAAD) is a malignant disease with poor prognosis, and the cure rate for this disease is only 9%, moving to be the third principal cause of cancer death; if untreated, the median survival was only 3 months of those patients with metastatic disease [1]. Thus, it is very urgent to search for more therapeutic targets for PAAD.

Long noncoding RNAs (lncRNAs) are a ribonucleic acid molecule with a length of more than 200 nucleotides, which does not have any protein coding function and participates in various cellular processes. An accumulating body of research indicates that the dysregulation of lncRNA expression is implicated in many kinds of cancer [2], including proliferation, apoptosis, migration, and invasion [3–5]. Moreover, lncRNAs can also act as diagnostic or prognostic markers in a range of cancer types, for an example, hepatocellular carcinoma and prostate cancer [6–8].

Epithelial-mesenchymal transition plays a prominent role in the formation of body plan and the differentiation of multiple tissues and organs. It is defined as the phenotypic transition from an epithelial state to a mesenchymal state in the morphological cell program. Study reveals that EMT can not only cause organ fibrosis but also promote carcinoma progression [9]. It involves a complex network including epigenetic modifications, transcriptional control, alternative splicing, protein stability, and subcellular localization [10–12]. Given that EMT are documented in many cancer cell models, the importance of EMT in cancer progression and the correlation in cancer tissues are still a matter of controversy. A multitude of researches have proven the invasiveness of PAAD associated with the EMT [13–16], while during the PAAD progression, how it works in an lncRNA-dependent way is unclear.

In our research, relying on The Cancer Genome Atlas (TCGA) dataset (*n* = 146) and the International Cancer Genome Consortium (ICGC) dataset (*n* = 92) and through the bioinformatics and statistical analysis of the PAAD patient data, the prognostic significance of the EMTlnc is determined. The results showed that 33 EMTlnc had prognostic value for PAAD patients in these two datasets. What is more, on the strength of the ability of 33 EMTlnc, we constructed an EMT-dependent predictive sign of lncRNA (EMT-LPS) that can predict the OS of PAAD patients. In the meantime, we found that in patients with PAAD, there exist different prognoses between low-risk and high-risk subgroups, and tumor features are more common in high-risk subgroups. Finally, we establish an accurate nomogram to predict the OS for PAAD patients, and a ceRNA network to search for target miRNA and mRNA was built.

## 2. Materials and Methods

### 2.1. Raw Data Acquisition

For the training set, mRNA expression files (Fragments Per Kilobase of transcript per Million mapped reads (FPKM) normalized) and the corresponding clinicopathological data were obtained from the Genomic Data Commons Data Portal (https://portal. http://gdc.cancer.gov/). For the validation set, the RNA-seq profile and associated clinicopathological data were downloaded from UCSC Xena Database (http://xena.ucsc.edu/) and the International Cancer Genome Consortium Data Portal (https://dcc.icgc.org/). For the purpose of reducing statistical bias, we excluded the PAAD patients with missing OS values or OS < 30 days. Finally, 146 PAAD patients from TCGA were selected to construct the EMT-LPS, and 92 PAAD patients from ICGC were included to test the EMT-LPS. Then, the FPKM data underwent a log2 transformation. The gene annotation file “gencode.v22.annotation,” which was downloaded from TCGA database, was utilized to transform ENSEMBL ID to GENE SYMBOL. On the basis of the GENCODE website (https://www.gencodegenes.org/human/), 9 types of transcripts (3prime_overlapping_ncRNA, antisense, bidirectional_promoter_lncRNA, lincRNA, macro_lncRNA, non_coding, processed_transcript, sense_intronic, and sense_overlapping) were defined as lncRNAs. The ENSEMBL ID with the max average expression level was used to represent the expression level of this gene when there were multiple ENSEMBL ID annotated to the same gene symbol. Ultimately, in TCGA cohort, there were 14805 lncRNAs and 19712 mRNAs, and in the ICGC cohort, the data were 1440 lncRNAs and 15146 mRNAs.

### 2.2. EMTlnc Acquisition by GSVA and WGCNA

The HALLMARK_EPITHELIAL_MESENCHYMAL_TRANSITION pathway, which contained 200 EMT-related genes, was obtained from the package “msigdbr” [17, 18]. The EMT pathway values for each PAAD patient were calculated using gene set variation analysis (GSVA) in TCGA cohort. Then, weighted gene coexpression network analysis (WGCNA) was applied to find significantly relevant lncRNA binding with lncRNA modules and find the relationship between the modules. Here, to provide a scale-free network, 50 were set to minimum cut size, with cut height = 0.3 combined with similar height modules. With different lncRNA modules marked with different colors, the gray modules represent lncRNA that cannot be combined; then, we applied the Pearson correlation analysis to evaluate the correlation of lncRNA and EMT values in each module. Finally, the lncRNAs in the model with abs(cor) > 0.5 and *p* value < 0.05 were defined as EMTlnc.

### 2.3. Establishment and Verification of EMT-LPS

The univariate Cox was used to choose prognostic EMTlnc in both TCGA cohort and ICGC cohort based on EMTlnc. Through taking intersection, 33 lncRNAs with *p* < 0.05 were defined as shared prognostic EMTlnc. Then, we used the LASSO Cox regression to select the significant prognostic lncRNAs and construct EMT-LPS involved 11 EMTlnc by the “glmnet” package [19] in R. Here, to facilitate parameter selection, the“10-fold cross-validation” approach was applied. The risk score of TCGA cohort and ICGC cohort patients was calculated as the following formula:

risk score = ∑_*i*=1_^*n*^Coef(*i*)∗*x*_*i*_, 

where Coef(*i*) is the estimated regression coefficient obtained from LASSO Cox regression analysis and *χ*_*i*_ is the expression value of each selected EMTlnc. PAAD patients were classified into low-risk and high-risk groups based on the median risk score. The OS results were then compared using the log-rank test and Kaplan-Meier survival analysis. To verify the accuracy of the identified EMT-LPS, the receiver operating characteristic (ROC) curve analysis in the package “Survival ROC” was applied [20].

### 2.4. Stratification Analysis

The entire TCGA cohort was stratified by age (≥60 (*n* = 95) or <60 (*n* = 43)), gender (female (*n* = 60) or male (*n* = 78)), grade (G1+G2 (*n* = 93), G3+G4 (*n* = 45)), and TNM stages (T1+T2 (*n* = 4), T3+T4 (*n* = 134), N0 (*n* = 38), N1 (*n* = 100)), while the ICGC cohort was divided into different subgroups (age (≥60 years (*n* = 67) or <60 years (*n* = 21)), gender (female (*n* = 47) or male (*n* = 41)), and TNM stages (T1+T2 (*n* = 3), T3+T4 (*n* = 85), N0 (*n* = 30), N1 (*n* = 58))). We applied the Wilcoxon rank sum test to compare the risk score of different stratification cohorts with the package “ggpubr” (https://cran.r-project.org/web/packages/ggpubr). The risk score formula obtained in TCGA cohort was applied to calculate the risk score of each PAAD patient in each stratified cohort and then divide them into the low-risk group and high-risk group. By Kaplan-Meyer survival analysis, the differences of OS results between the low-risk group and high-risk group were compared by the logarithmic rank test.

### 2.5. Principal Component Analysis (PCA) and Nomogram Construction

Based on the expression of 200 EMT-related genes in the HALLMARK_EPITHELIAL_MESENCHYMAL_TRANSITION pathway, we used PCA to evaluate the differences between low-risk and high-risk subgroups and constructed a nomogram using the R package “RMS” [21] to evaluate the 1-, 2-, and 3-year survival possibility of patients in the cohorts. We then generated the calibration curve of the nomogram and evaluated the consistency between the predicted value and the actual observed value through the rms package.

### 2.6. Construction of the ceRNA Network

On the basis of the threshold ∣log2 (fold change) | >1 and *p* < 0.05, the differentially expressed genes (DEGs) between low-risk and high-risk subgroups were identified by the Wilcoxon rank sum test through TCGA cohort. 11 target miRNAs of EMTlnc in the miRcode database (http://www.mircode.org/) were predicted and analyzed by using the Perl programming language. Then, in miRTarBase (http://mirtarbase.mbc.nct.edu.tw/PHP/index.php), miRDB (http://mirdb.org/), and TargetScan databases (http://www.targetscan.org/), we found the shared target mRNA of these miRNAs, crossed with DEGs, and got differential expression. Finally, we drew the ceRNA network through the “Cytoscape” software [20].

### 2.7. Enrichment Analysis

We inputted differentially expressed genes between low-risk and high-risk subgroups and targeted mRNAs differentially expressed in the ceRNA network into the “Metascape” website (http://metascape.org/) for function and pathway enrichment analysis, including typical pathway, Reactome gene set, Gene Otology (GO) biological process, and Kyoto Gene and Genome Encyclopedia pathway (KEGG pathway). In addition, in order to study which tumor features are more common in high-risk subgroups, GSEA software (http://software.broadinstitute.org/gsea/index.jsp) was used.

### 2.8. Statistical Analysis

In this study, the statistical analysis of all datasets is carried out with the R programming language (4.0.0). Kaplan-Meier, log rank test, and univariate Cox regression were applied for survival analysis based on the expression of EMT-related lncRNAs contained in EMT-LPS. Univariate and multivariate Cox regression analyses were used to evaluate the independent predictive value of EMT-LPS for OS prognosis.

## 3. Results

### 3.1. Identification of EMTlnc in PAAD Patients

First, 14805 lncRNAs from TCGA dataset and 1440 lncRNAs from the ICGC dataset were identified for the next analysis. Then, we analyzed the genes related to the EMT pathway in TCGA PAAD samples by GSVA and got the GSVA variation. After applying WGCNA on the database of PAAD patients in TCGA, the coexpression network by WGCNA analysis showed that the EMT-related lncRNA models in PAAD samples were grouped into 48 models, containing MElightcyan and MEturquoise models with abs(cor) > 0.5 and *p* value < 0.05, which were both included into ulterior analysis in this research. The lncRNAs in those two models were defined as EMTlnc.

**Figure 1:**
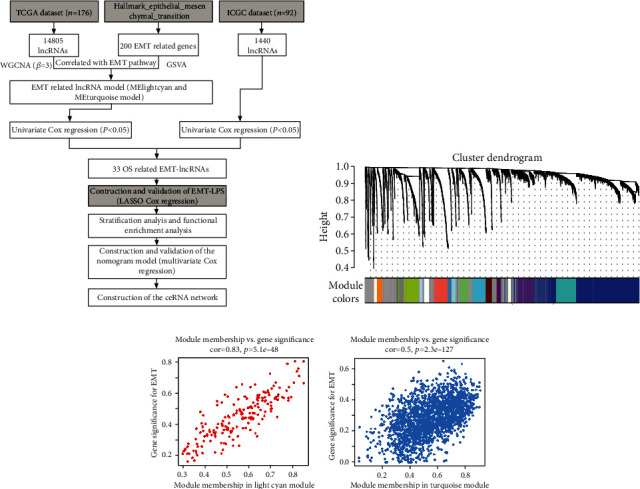
(a) Research flowchart. (b) WGCNA of lncRNAs in PAAD samples. Gene clustering tree (dendrogram) obtained by hierarchical clustering based on adjacency-based dissimilarity. (c) Through the Pearson correlation analysis, the correlation between the membership in the light cyan module and the membership of the characteristic genes in the light cyan module in the EMT pathway is determined. Cor: correlation coefficient. (d) Through the Pearson correlation analysis, the correlation between the membership of the turquoise module and the membership of the autophagy pathway of the turquoise module characteristic gene is determined. Cor: correlation coefficient.

With the combination of the prognostic information, we implement univariate Cox regression (*p* < 0.05) in two datasets, screening out the prognostic reactions associated with EMT from the EMTlnc. Ultimately, in these two datasets, by cross-analysis, we found that EMTlnc was significantly correlated with the OS of PAAD patients. The workflow is shown in Figure 1(a), and WGCNA in the PAAD sample is shown in Figure 1(b). The Pearson correlation analysis showed the correlation between the membership in the light cyan module and membership of characteristic genes in the light cyan module in the EMT pathway and the Pearson correlation analysis showed the correlation between the membership in the turquoise module and characteristic genes in the turquoise module in the EMT pathway as shown in Figures 1(c) and 1(d), respectively. The univariate Cox analysis results of 33 EMTlnc are shown in Table 1.

### 3.2. Construction of the EMT-LPS in TCGA Dataset and Validation of the EMT-LPS in the ICGC Dataset

**Figure 2:**
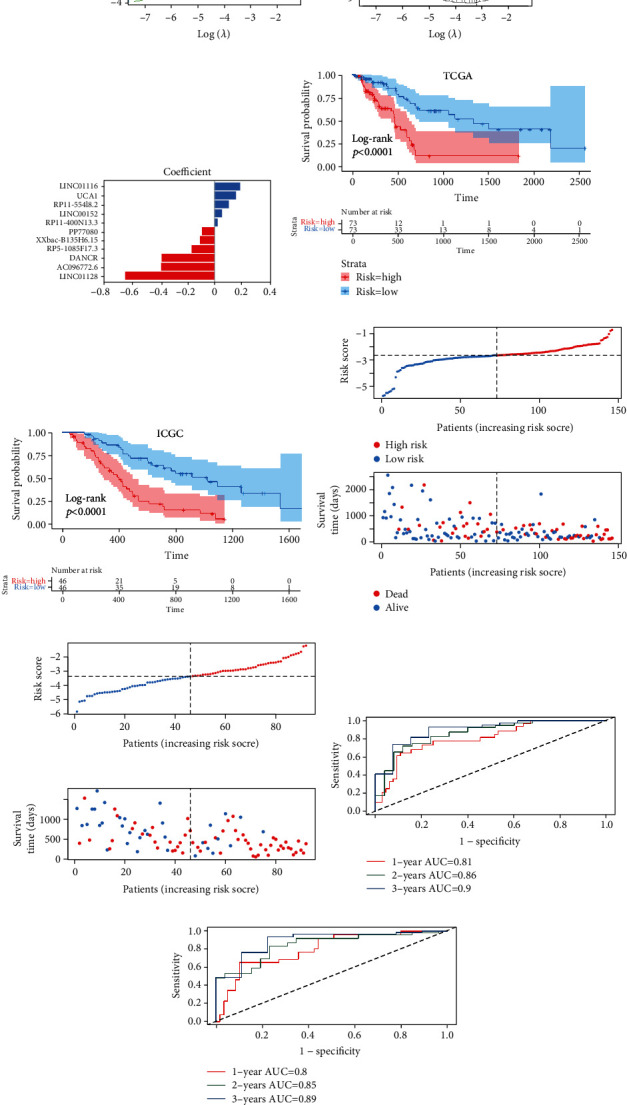
Construction of EMT-LPS in TCGA dataset and validation in the ICGC dataset. (a–c) LASSO regression was performed to calculate the minimum criteria (a, b) and coefficients (c). (d, e) Kaplan-Meier survival curve of low-risk and high-risk groups divided by the cutoff value in TCGA dataset (d) and ICGC dataset (e), respectively. *p* values were obtained by the log-rank test. Risk scores and distribution of survival status of 11 EMT lncRNAs selected from TCGA dataset (f) and ICGC dataset (g), respectively. (h, i) The ROC for the prognosis prediction of the signature at 1/2/3 years of overall survival (OS) in TCGA dataset (h) and ICGC dataset (i), respectively.

In TCGA cohort, on the basis of 33 EMT-related prognostic lncRNAs, we performed a LASSO Cox analysis to build the EMT-LPS for the prediction of PAAD patients' OS, and coefficients containing 11 EMT-LPS were generated (Figures 2(a) and 2(b)). The EMT-LPS included 11 lncRNAs, and the risk assessment is calculated from the coefficient of each lncRNA (Figure 2(c)). Then, according to the median risk score, the patients were divided into low-risk and high-risk subgroups. The Kaplan-Meyer survival curve showed that PAAD patients with poor clinical outcomes usually have higher risk scores, which means lower OS incidence and shorter OS time (Figure 2(d)).  Figure 2(f) plots the risk score and survival status. The ROC curve (Figure 2(h)) showed that EMT-LPS had a strong ability to predict OS in TCGA cohort (AUC = 0.86, 0.86, and 0.9 for 1, 2, and 3 years, respectively).

To verify the predictive ability of EMT-LPS, we calculated the same formula for these patients in the ICGC cohort. Based on the average risk score, we divided PAAD patients into low-risk and high-risk groups. The results were consistent with TCGA dataset: in the ICGC dataset, patients with higher risk scores had lower OS rates and shorter OS time (Figure 2(e)).  Figure 2(g) shows the assessment of risk and survival status distribution, which indicated that the total survival time and death status were shorter in patients with the higher risk score. ROC analysis also showed that EMT-LPS has a powerful predictive value for PAAD patients (AUC = 0.895, 0.85, and 0.89 for 1, 2, and 3 years, respectively; Figure 2(i)). These results comprehensively implied that the EMT-LPS had a strong and stable OS prediction ability.

### 3.3. Prognostic Analysis of the Eleven EMTlnc

**Figure 3:**
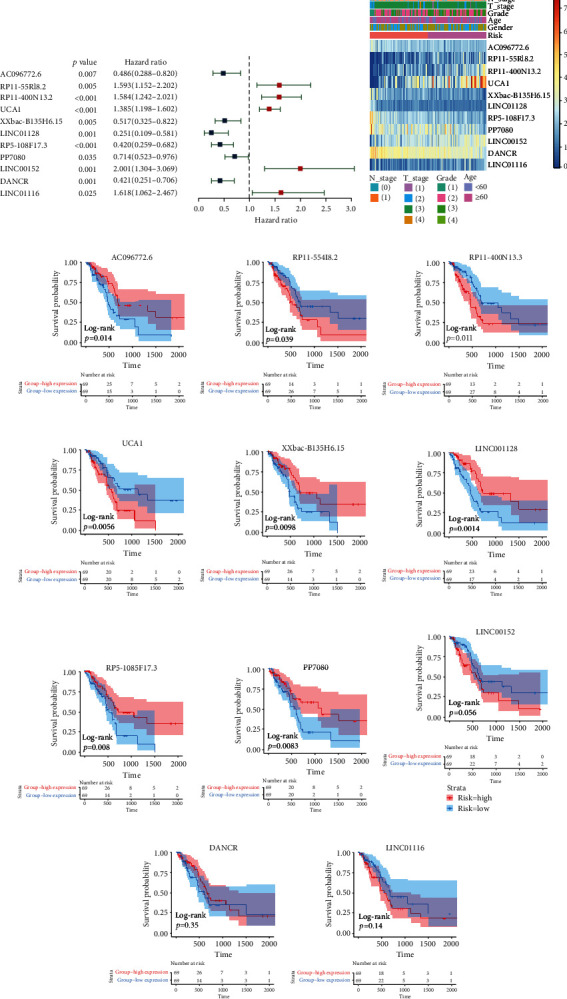
Prognostic analysis of the elven EMT-related lncRNAs in TCGA. (a) Forest plot of 11 EMT-related lncRNAs, including their predictive properties. (b) Heatmap of the eleven EMT-related lncRNAs and related clinicopathological features in TCGA dataset. (c–m) Kaplan-Meier curves showing different EMT-related lncRNAs' expression levels and overall survival times in patients.

There were eleven EMTlnc in the EMT-LPS, and to evaluate their prognostic roles, univariate Cox regression analysis was performed. In the forest plot, we can see that PP7080, xxbac-B135H6.15, RP5-1085F17.3, DANCR, AC096772.6, and LINC01128 are protective factors (HR: hazard ratio < 1), while LINC01116, UCA1, and RP11-400N13.3 are risk factors in EMT patients (HR < 1, Figure 3(a)). Heatmap (Figure 3(b)) indicates that with the increase in the risk score, RP11-55418.2, RP11-400N13.3, UCA1, LINC00152, and LINC01116 expression levels were also increased; however, the expression of the AC096772.6, xxbac-B135H6.15, LINC01128, RP5-1085F17.3, PP7080, and DANCR was decreased. Their expression levels were also connected with PAAD's clinicopathological features, including N_stage, T_stage, gender, age, and WHO grade (Figure 3(b)). Kaplan-Meier survival curves verified the high expression of AC096772.6, xxbac-B135H6.15, LINC01128, RP5-1085F17.3, and PP7080, and the low expression of RP11-55418.2, RP11-400N13.3, UCA1, LINC00152, DANCR, and LINC01116 was related to better OS in TCGA dataset (Figures 3(c)–3(m)). In the ICGC dataset, the heatmap (Supplementary Figure S2C) also showed that RP11-55418.2, RP11-400N13.3, UCA1, LINC00152, and LINC01116 expression increased with the increase in the risk score, while the expression of the AC096772.6, xxbac-B135H6.15, LINC01128, RP5-1085F17.3, PP7080, and DANCR decreased with the increasing risk score. Their expression levels were connected with PAAD's clinicopathological features, such as N_stage, T_stage, gender, and age.

### 3.4. Stratification Analysis of the EMT-LPS

**Figure 4:**
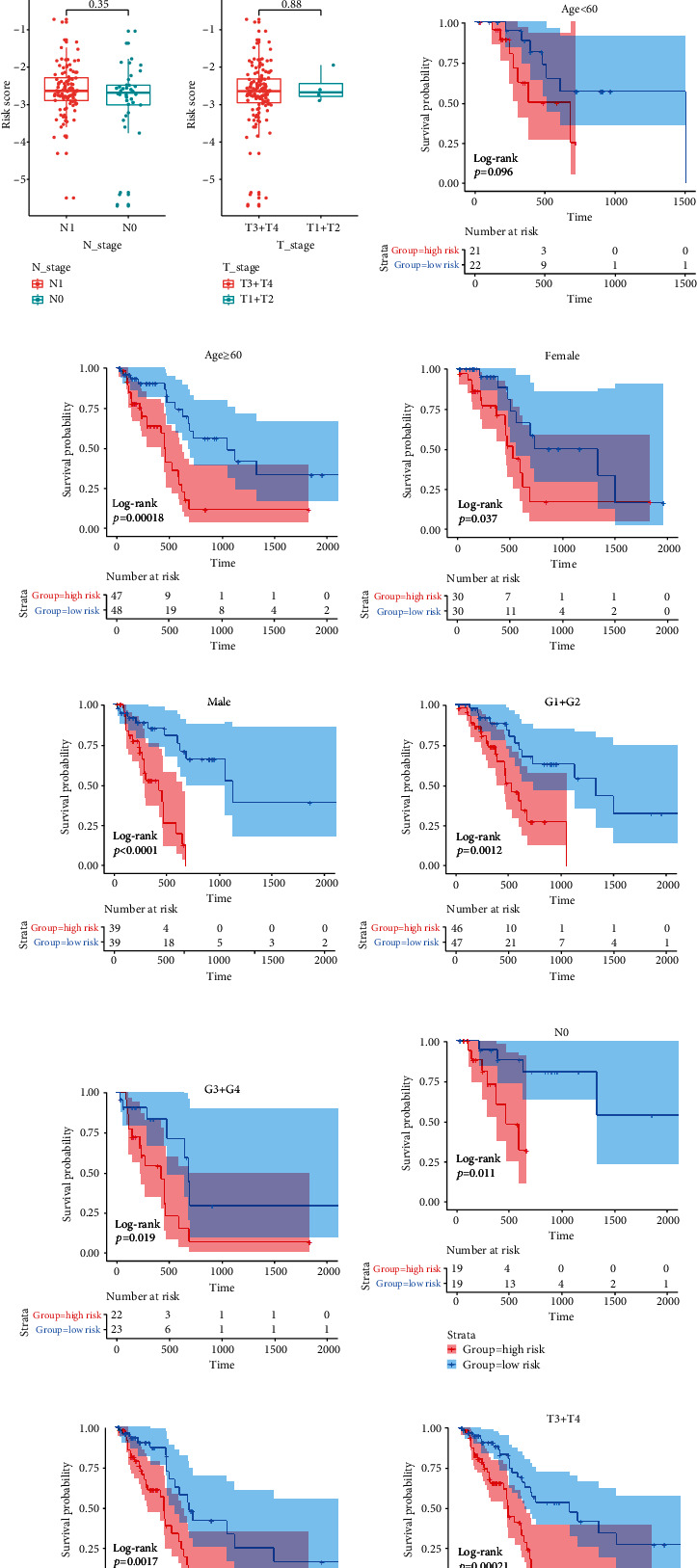
Stratification analysis of the EMT-LPS in TCGA patients with different clinical characteristics. (a–e) Patients with different clinicopathological features had different levels of risk scores. (g–l) EMT-LPS acquired prognostic ability in different subgroups of PAAD patients.

We tried to determine if the clinicopathological features were related to the risk score or not. In TCGA dataset, the results showed the PAAD patients whose WHO grade at III+IV were in a higher risk scores, but the risk score had no association with age, gender, N_stage, and T_stage (Figures 4(a)–4(e)). To have a better assessment on the prognostic ability of EMT-LPS, we conducted a stratification analysis to verify if it retains the ability to predict OS in different subgroups. On the contrary to the PAAD patients with lower risk, patients with higher risk had worse OS when they were ≥60 years old (Figures 4(f) and 4(g)). In the same way, we proved that EMT-LPS can predict the OS of female or male PAAD patients (Figures 4(h) and 4(i)), patients with grade I+II or grade III+IV, and patients with TNM stage N0, N1, or T3+T4 (Figures 4(j)–4(n)). In the ICGC dataset, the results showed that PAAD patients with N1_stage, T3_stage, and T4_stage had higher risk scores (Supplementary Figure S2D–G). These data testified that the EMT-LPS might be a potential predictor of PAAD patients.

### 3.5. Principal Component Analysis

We conducted principal component analysis (PCA) on the basis of the expression value of the 200 EMT-related genes to evaluate the differences between the low-risk and high-risk groups (Supplementary Figure S5A, B). The results showed that the low-risk and high-risk patients were distributed in distinct directions in both TCGA and ICGC datasets. These results may suggest that different risk subgroups have different EMT statuses.

### 3.6. Pathway and Process Enrichment Analysis and Gene Set Enrichment Analysis (GSEA)

We identified 710 differentially expressed genes (DEGs) (∣log2 (fold change) | >1 and *p* < 0.05) between two subgroups in TCGA cohort to study the potential biological process and pathway concerning molecular heterogeneity between the low-risk and high-risk subgroups. These DEGs were mainly focused on the following aspects: chemical synaptic transmission, neuronal system, membrane potential regulation, behavior, projection morphogenesis of plasma membrane-binding cells, and GABAergic synapse (Supplementary Material S1A). GSEA showed that the interferon alpha response and the interferon gamma response (two tumor hallmarks) were enriched in the high-risk subgroup (Supplementary Material S1B, C). These results may be helpful to elucidate the cell biological effects of EMT-LPS.

### 3.7. EMT-LPS Was an Independent Prognostic Factor in PAAD Patients

We used univariate and multivariate Cox analyses to evaluate whether EMT-LPS was an independent prognostic factor in PAAD patients. The former showed that EMT-LPS was significantly correlated with OS based on TCGA dataset (hazard ratio (HR): 3.832, 95% CI: 2.435-6.030, *p* < 0.001; Supplementary Material S2A). The latter showed that EMT-LPS was an independent predictor of OS (HR: 3.573, 95% CI: 2.248-5.681, *p* < 0.001; Supplementary Material S2A). This conclusion was verified in the ICGC dataset. It was confirmed that EMT-LPS was an independent predictor of OS of PAAD patients in the ICGC dataset (univariate: HR: 2.667, 95% CI: 1.923-3.697, *p* < 0.001; multivariate: HR: 2.787, 95% CI: 1.945-3.94, *p* < 0.001; Supplementary Material S2B). The results above proved that EMT-LPS was an independent prognostic indicator and of great significance for clinical prognosis evaluation.

### 3.8. Construction and Validation of the Nomogram Based on EMT-LPS

By using risk status (based on EMT-LPS), gender, age, T_stage, N_stage, and WHO grade in TCGA dataset, we constructed a nomogram to create a clinically applicable quantitative tool to predict the OS of PAAD patients and tested in the ICGC dataset (Supplementary Material S2C). Calibration plots indicated that 1-, 2-, and 3-year OS ratios are completely consistent with the predicted ratios in these two cohorts (Supplementary Material S3A-C and Supplementary Material S4B-D, respectively). Then, we used the time-dependent ROC curves to evaluate the predictive ability of the nomogram and other predictors in TCGA (risk score, gender, age, T_stage, N_stage, and WHO grade, Supplementary Material S3D-F and Supplementary Figure S1E-G, respectively); the results showed that the nomogram had excellent accuracy in 1-, 2-, and 3-year OS (AUC of TCGA was 0.79, 0.83, and 0.86; AUC of ICGC was 0.8, 0.84, and 0.89). These data suggested that the nomogram has strong and stable capabilities to predict the OS for PAAD patients.

### 3.9. Construction of the ceRNA Network and Functional Enrichment Analysis

Based on the EMTlnc, we established a ceRNA network to have a deeper insight on how EMTlnc regulates mRNA expression by sponging miRNAs in PAAD. Two of 12 lncRNAs were extracted from the miRcode database, and 13 pairs of interactions between 2 lncRNAs and 34 miRNAs were identified. Then, we used three databases (miRTarBase, miRDB, and TargetScan) to search for target mRNAs on the basis of the 34 miRNAs, and 1539 mRNAs were identified in these three databases.

**Figure 5:**
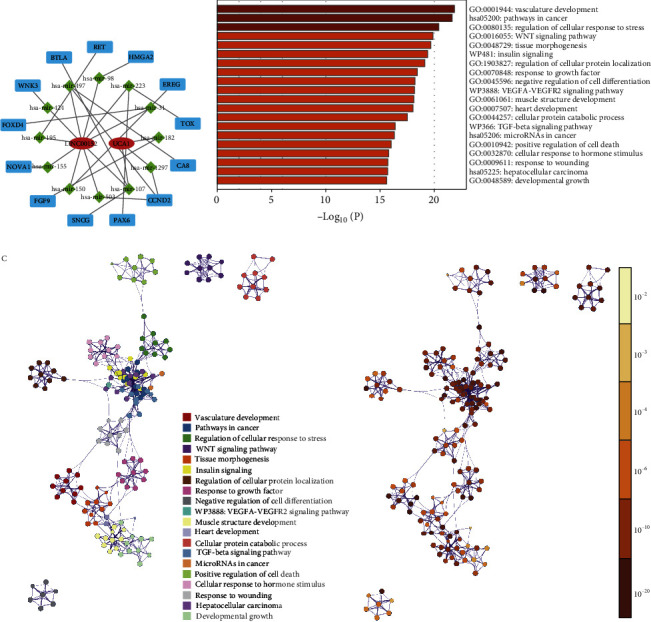
(a) Results of network and functional enrichment analysis of ceRNA. The ceRNA network consists of two EMT-related lncRNAs (red), target miRNAs (green), and mRNAs (blue). (b) Heatmap of enriched terms across the 929 mRNAs and the network of enriched terms, colored according to the *p* value. (c) Cluster ID (nodes with the same cluster ID are typically close to each other) and (d) *p* value (terms with more genes tend to have a higher *p* value).

Furthermore, these target mRNAs were crossed with DEGs to obtain differentially expressed target mRNA. Finally, two lncRNAs, twelve miRNAs, and thirteen mRNAs were included in the ceRNA network (Figure 5(a)). Additionally, functional enrichment in the network tool using 1539 mRNA targets revealed that these genes are enriched in vascular system development, pathway in cancer, regulation of cellular response to stress, Wnt signaling pathway, tissue morphogenesis, insulin signaling, regulation of cellular protein localization, response to growth factor, and negative regulation of cell differentiation (Figures 5(b)–5(d)). These data can provide some clues for us to discover the potential functionality of EMTlnc in PAADs.

## 4. Discussion

In this study, 268 PAAD patients in TCGA and ICGC datasets were included to explore the prognostic significance of EMTlnc. In these two datasets, 33 EMTlnc were proven to have predictive value and 11 EMTlnc among them were used to establish EMT-LPS to predict PAAD OS. On the basis of the median risk score, we divided PAAD patients into low-risk and high-risk subgroups and found that the latter group had worse clinical outcomes, richer tumor characteristics, and exact malignant-related pathways. Multivariate Cox regression analysis revealed the EMT-LPS was an independent risk factor for OS.

In addition, we set up a nomogram of EMT-LPS combined with gender, age, and World Health Organization grade, T_stage and N_stage, which has a strong predictive ability for OS of PAAD patients on TCGA and ICGC datasets. Finally, we established a ceRNA network that includes two EMTlnc, twelve miRNAs, and thirteen mRNAs for observing the latent functions of these EMTlnc.

More and more evidences show that lncRNAs can coordinate multiple cell processes by regulating EMT in different types of cells. MALAT1 and lnc-ATB can stimulate the EMT process by competitively binding miR-503 and miR-200c and then promote silica-induced pulmonary fibrosis [22, 23]. In EMT of the breast, bladder, and nasopharynx, lncRNA ROR can regulate various signal pathways [24–26]. More importantly, by inducing EMT to accelerate the growth and progression of cancer, hypoxia can enhance the mutual movement of lncRNA UCA1 into the exon-mediated bladder cancer cells [27].

Studies had revealed that EMT had impact on cancer invasion and progression, and lncRNAs may play the role of ceRNAs, targeting EMT regulators so as to influence the aggressive progression of the tumor. Liu et al. [28] found that TGFBI acts as the ceRNA of miR-21 and FN1 acts as miR-200c to regulate EMT. What is more, the richness of ceRNA can determine the reversibility of EMT. By the way, it was reported that the mutations in BRCA2 significantly increase the risk of pancreatic cancer [29]; whether it is associated with EMT is a question that needs to be further explored. miR-330 might be of potential therapeutic value for pancreatic cancer pain because it participated in the genesis of pancreatic carcinoma-induced pain hypersensitivity [30]; perhaps we can explore the mechanism of pancreatic cancer pain from the perspective of EMT. Here, we implemented a ceRNA-based functional enrichment analysis and found that genes were mainly enriched in vascular system development, pathway in cancer, cell stress response regulation, Wnt signaling pathway, and some other pathways. Taking all these evidences into consideration, we bet that EMT is targeted at lncRNAs, and to identify potential prognostic markers or therapeutic targets, we should better place more emphasis on the interactions and functions of lncRNAs and EMT.

From 268 PAAD patients, we identified 33 EMT-related prognostic lncRNAs, eleven of which were included in the EMT-LPS. Yang et al. uncovered that RP11-400N13.3 reacts as an oncogenic lncRNA in colorectal cancer, and by modulating the miR-4722-3p/P2RY8 axis, it can accelerate colorectal cancer progression [31]. LINC00152 was firstly found overexpressed in gastric cancer and acted as an oncogene in gliomas and liver, lung, and colorectal cancer [32–34]. It can promote cell proliferation and invasion capability of these cancer cells by targeting GFR, EZH2, miR-16, and miR-139-5p [35–39]. Wang et al. confirmed that the overexpression of LINC01116 was related to the clinicopathological characteristics and survival in glioma patients and can accelerate tumor proliferation and neutrophil recruitment by regulating IL-1*β* [40]. Li et al. found that LINC01128 can resist acute myeloid leukemia by regulating miR-4260/NR3C2 [41]. Tang et al. reported that miR-135a can downregulate DANCR by regulating the downstream of NLRP3 in pancreatic cancer [42], which is consistent with our results.

Some of the 11 lncRNAs have been reported to be related to cancer progression, but little is reported about PAAD, let alone how lncRNAs interact with EMT-related genes. Hence, we hope that our results will help determine the predictive responses that EMT regulators may target, thus providing insight into its potential role in carcinogenesis and PAAD development.

In a word, this study included two PAAD datasets (TCGA and ICGC datasets) through which our results have been obtained and validated, but there also are some limitations. In the future, we should use more independent PAAD cohorts to verify the established prognostic EMTlnc, design new drugs or strategies to manage PAAD, and advocate more research to reveal the specific mechanism and genes of PAAD regulating EMT.

## 5. Conclusions

EMT-LPS played an important role in PAAD's carcinoma progression and may help us in choosing the way of prognosis assessment and provide some clues to design the new drugs for PAAD.

## Figures and Tables

**Table 1:** The thirty-three EMT-related prognostic lncRNAs.

EMT-related lncRNAs	TCGA				ICGC			
	HR	HR.95L	HR.95H	*p* value	HR	HR.95L	HR.95H	*p* value
AC017002.1	2.9985	1.0379	8.6629	4.25*E* − 02	1.4835	1.0744	2.0483	1.66*E* − 02
AC093850.2	1.2967	1.0605	1.5855	1.13*E* − 02	1.1653	1.0152	1.3376	2.96*E* − 02
LINC00152	1.9583	1.3243	2.8959	7.60*E* − 04	1.9065	1.2703	2.8615	1.84*E* − 03
LINC01116	1.7473	1.1670	2.6161	6.73*E* − 03	1.5378	1.1805	2.0033	1.42*E* − 03
MIR4435-1HG	2.3464	1.4175	3.8839	9.10*E* − 04	2.3326	1.4546	3.7404	4.39*E* − 04
RP11-274H2.3	5.8172	1.4789	22.8821	1.17*E* − 02	1.3529	1.0352	1.7681	2.69*E* − 02
RP11-400 N13.3	1.6652	1.3155	2.1079	2.24*E* − 05	1.2304	1.0762	1.4068	2.41*E* − 03
RP11-417E7.1	1.5019	1.0033	2.2482	4.82*E* − 02	1.3728	1.1670	1.6149	1.31*E* − 04
RP11-554I8.2	1.6514	1.2114	2.2512	1.51*E* − 03	1.1728	1.0457	1.3153	6.46*E* − 03
UCA1	1.4171	1.2289	1.6341	1.62*E* − 06	1.3475	1.1798	1.5391	1.10*E* − 05
AC009506.1	0.2309	0.0902	0.5911	2.24*E* − 03	0.6498	0.4526	0.9330	1.95*E* − 02
AC096772.6	0.4648	0.2784	0.7762	3.40*E* − 03	0.5803	0.3373	0.9985	4.93*E* − 02
DANCR	0.3418	0.2100	0.5562	1.56*E* − 05	0.6598	0.4665	0.9331	1.87*E* − 02
FLJ37035	0.0148	0.0007	0.3109	6.69*E* − 03	0.7340	0.5878	0.9165	6.35*E* − 03
GS1-358P8.4	0.3759	0.2246	0.6289	1.95*E* − 04	0.3935	0.2300	0.6734	6.67*E* − 04
HNF1A-AS1	0.5779	0.4157	0.8034	1.11*E* − 03	0.7402	0.5912	0.9267	8.70*E* − 03
LINC00261	0.6925	0.5461	0.8780	2.41*E* − 03	0.8859	0.8096	0.9693	8.33*E* − 03
LINC01128	0.2883	0.1461	0.5691	3.37*E* − 04	0.3458	0.1789	0.6681	1.58*E* − 03
PART1	0.0495	0.0026	0.9468	4.59*E* − 02	0.8745	0.7691	0.9944	4.09*E* − 02
PP7080	0.6783	0.4953	0.9288	1.55*E* − 02	0.7559	0.5729	0.9974	4.79*E* − 02
PRKAG2-AS1	0.6626	0.4526	0.9702	3.44*E* − 02	0.7983	0.6602	0.9652	2.00*E* − 02
RP11-16P6.1	0.0868	0.0184	0.4096	2.02*E* − 03	0.6257	0.3945	0.9926	4.64*E* − 02
RP11-226 L15.5	0.4160	0.2031	0.8521	1.65*E* − 02	0.5727	0.3337	0.9830	4.31*E* − 02
RP11-244O19.1	0.1963	0.0790	0.4874	4.50*E* − 04	0.7268	0.5435	0.9720	3.14*E* − 02
RP11-384 L8.1	0.5399	0.3382	0.8617	9.78*E* − 03	0.6399	0.5037	0.8129	2.55*E* − 04
RP11-700H6.1	0.0668	0.0087	0.5136	9.31*E* − 03	0.8289	0.7284	0.9433	4.45*E* − 03
RP1-193H18.2	0.3373	0.1930	0.5895	1.36*E* − 04	0.7516	0.6052	0.9334	9.77*E* − 03
RP11-968O1.5	0.5038	0.2815	0.9017	2.10*E* − 02	0.7650	0.5933	0.9863	3.88*E* − 02
RP5-1033H22.2	0.6204	0.3941	0.9766	3.92*E* − 02	0.8018	0.6884	0.9338	4.50*E* − 03
RP5-1085F17.3	0.3766	0.2402	0.5904	2.07*E* − 05	0.5024	0.2875	0.8779	1.56*E* − 02
RP5-894A10.2	0.5887	0.3842	0.9020	1.49*E* − 02	0.6040	0.4265	0.8554	4.52*E* − 03
SLC25A25-AS1	0.6439	0.4690	0.8839	6.45*E* − 03	0.7420	0.5867	0.9383	1.27*E* − 02
XXbac-B135H6.15	0.4622	0.2895	0.7377	1.22*E* − 03	0.6998	0.5027	0.9740	3.44*E* − 02

Color-shaded lncRNAs were risky lncRNAs, and others were protective lncRNAs.

## Data Availability

The datasets during the current study are available from the corresponding author upon reasonable request.

## Supplementary Materials

Supplementary Materials Supplementary Material S1: functional analysis of 710 differentially expressed mRNA (DEms) between low-risk and high-risk subgroups. Supplementary Material S2: EMT-LPS was an independent prognostic factor for PAAD patients. Supplementary Material S3: construction of EMT-LPS calibration in TCGA dataset. Supplementary Material S4: nomogram and calibration based on EMT-LPS in the ICGC dataset. Supplementary Material S5: (A, B) Principal component analysis of EMT-LPS in TCGA and ICGC datasets. (C) Heatmap of the relationship between the expression levels of eleven EMT-related lncRNAs and clinicopathological features in the ICGC dataset. (D-G) Stratification analysis of EMT-LPS in ICGC patients with different clinical characteristics.
