# Supplementary material for: Identification of EMT-Related lncRNAs as Potential Prognostic Biomarkers and Therapeutic Targets for Pancreatic Adenocarcinoma
Source: J Oncol. 2022 Apr 11;2022:8259951. doi: 10.1155/2022/8259951 (PMC9015861; doi:10.1155/2022/8259951)
Supplement: Supplementary Materials — Supplementary Material S1: functional analysis of 710 differentially expressed mRNA (DEms) between low-risk and high-risk subgroups. Supplementary Material S2: EMT-LPS was an independent prognostic factor for PAAD patients. Supplementary Material S3: construction of EMT-LPS calibration in TCGA dataset. Supplementary Material S4: nomogram and calibration based on EMT-LPS in the ICGC dataset. Supplementary Material S5: (A, B) Principal component analysis of EMT-LPS in TCGA and ICGC datasets. (C) Heatmap of the relationship between the expression levels of eleven EMT-related lncRNAs and clinicopathological features in the ICGC dataset. (D-G) Stratification analysis of EMT-LPS in ICGC patients with different clinical characteristics. [file 8259951.f1.zip › Supplementary material S1.pdf]

Supplementary meterial S1

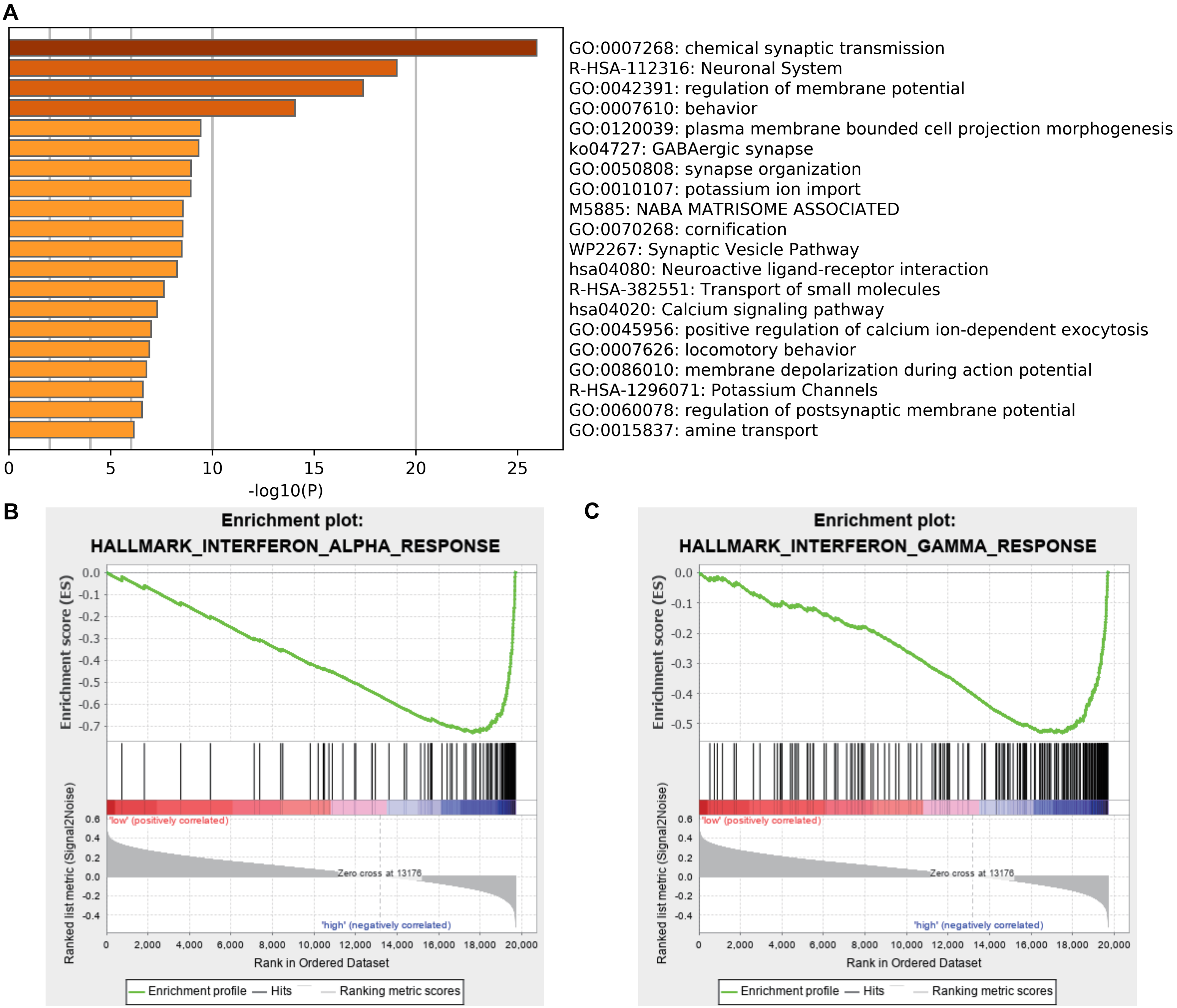

Upregulated in class	High risk	Upregulated in class	High risk
Enrichment Score (ES)	-0.7310267	Enrichment Score (ES)	-0.52983075
Normalized Enrichment Score (NES)	-1.8306823	Normalized Enrichment Score (NES)	-1.6644077
Nominal p-value	0.003968254	Nominal p-value	0.049822062
FDR q-value	0.16821192	FDR q-value	0.3240651
FWER p-Value	0.122	FWER p-Value	0.302
