# Supplementary figures and images for: Identification of EMT-Related lncRNAs as Potential Prognostic Biomarkers and Therapeutic Targets for Pancreatic Adenocarcinoma
Source: J Oncol. 2022 Apr 11;2022:8259951. doi: 10.1155/2022/8259951 (PMC9015861; doi:10.1155/2022/8259951)

# Supplementary material S2

A

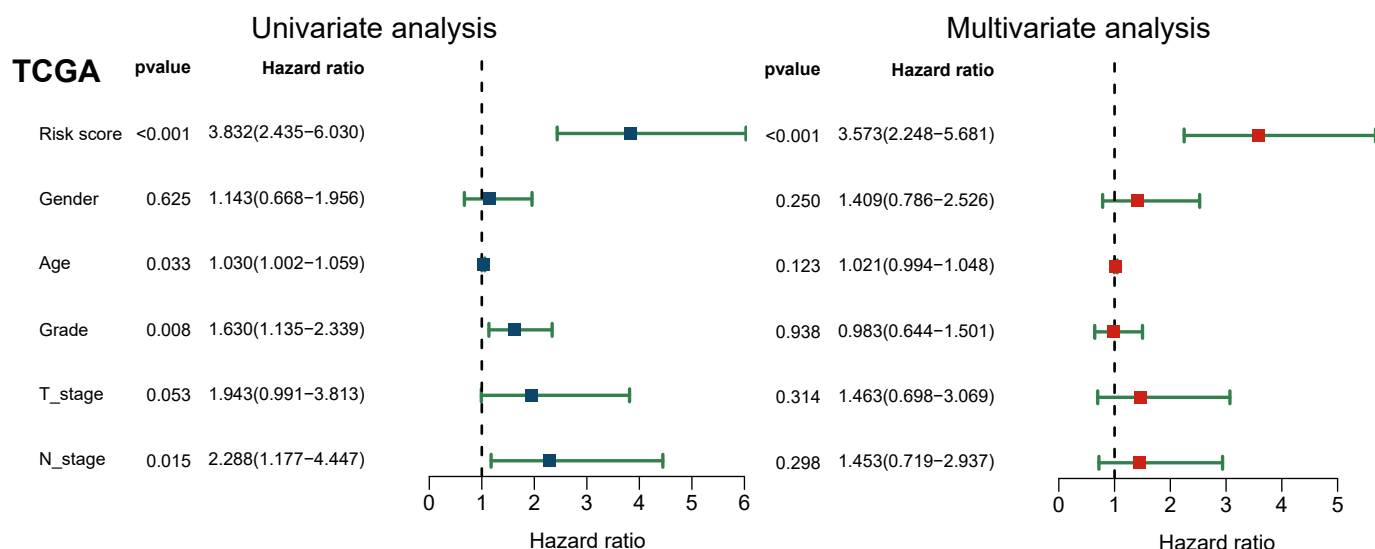

B

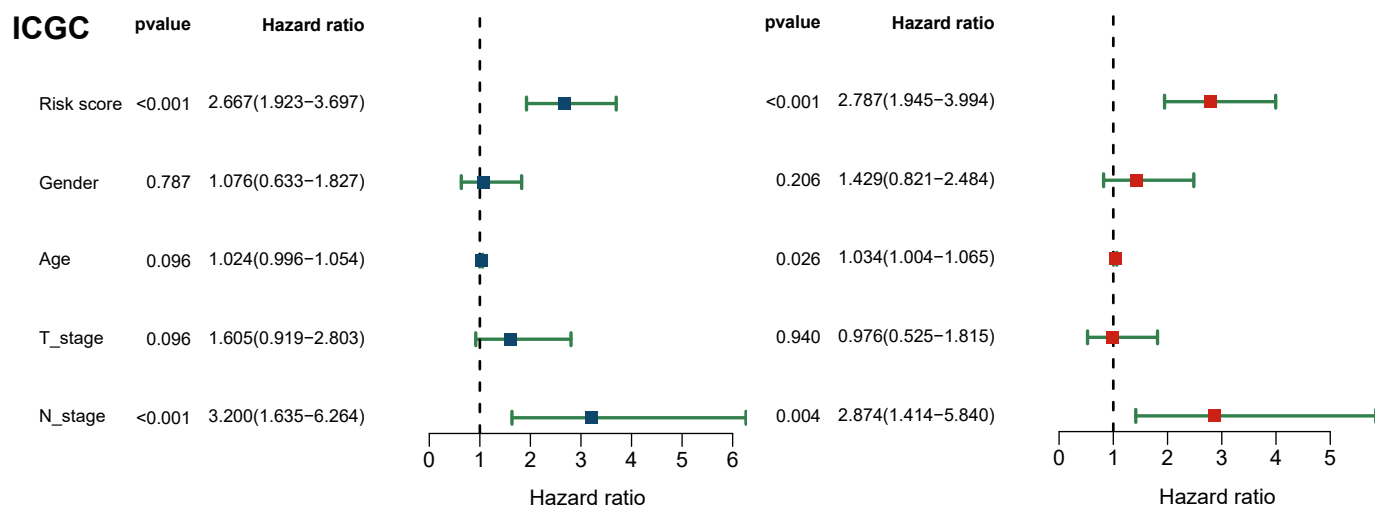

C

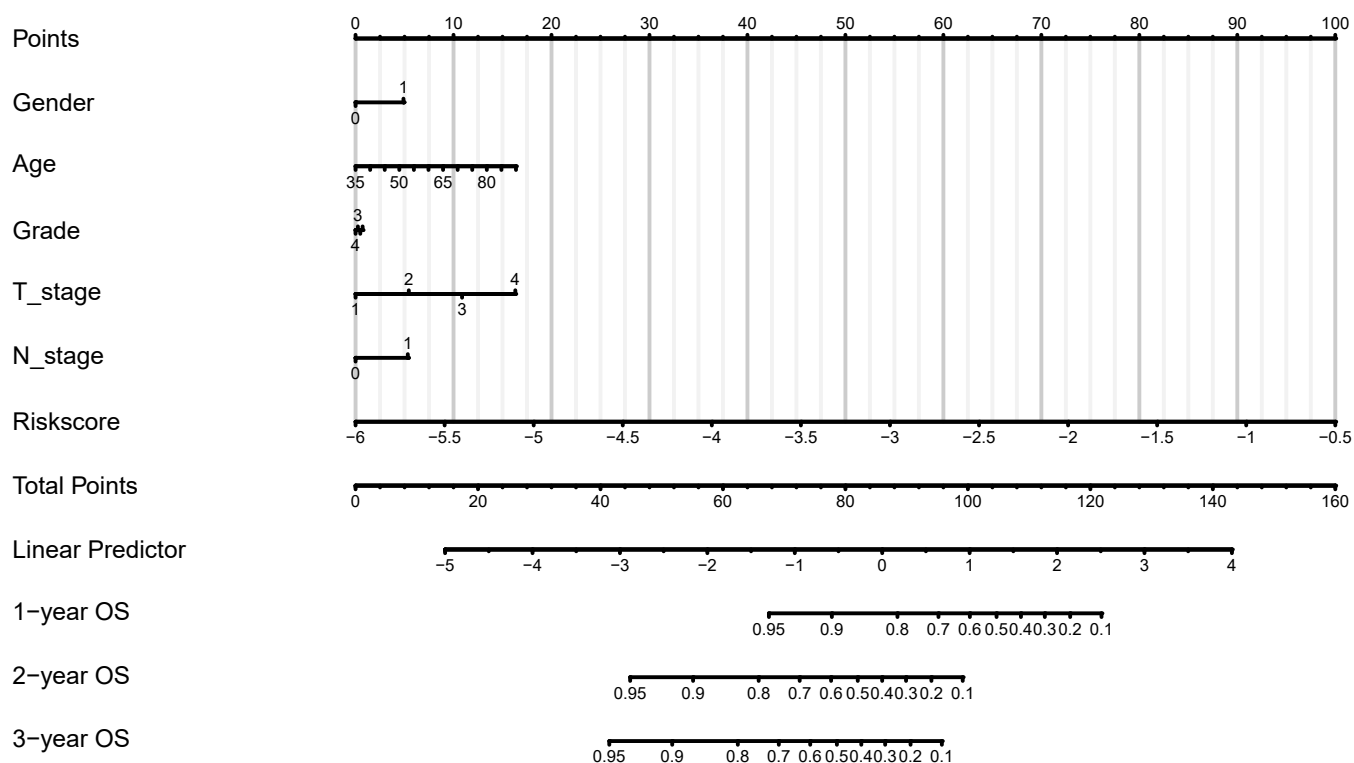

Supplement: Supplementary Materials — Supplementary Material S1: functional analysis of 710 differentially expressed mRNA (DEms) between low-risk and high-risk subgroups. Supplementary Material S2: EMT-LPS was an independent prognostic factor for PAAD patients. Supplementary Material S3: construction of EMT-LPS calibration in TCGA dataset. Supplementary Material S4: nomogram and calibration based on EMT-LPS in the ICGC dataset. Supplementary Material S5: (A, B) Principal component analysis of EMT-LPS in TCGA and ICGC datasets. (C) Heatmap of the relationship between the expression levels of eleven EMT-related lncRNAs and clinicopathological features in the ICGC dataset. (D-G) Stratification analysis of EMT-LPS in ICGC patients with different clinical characteristics. [file 8259951.f1.zip › Supplementary material S2.pdf]

# Supplementary material S3

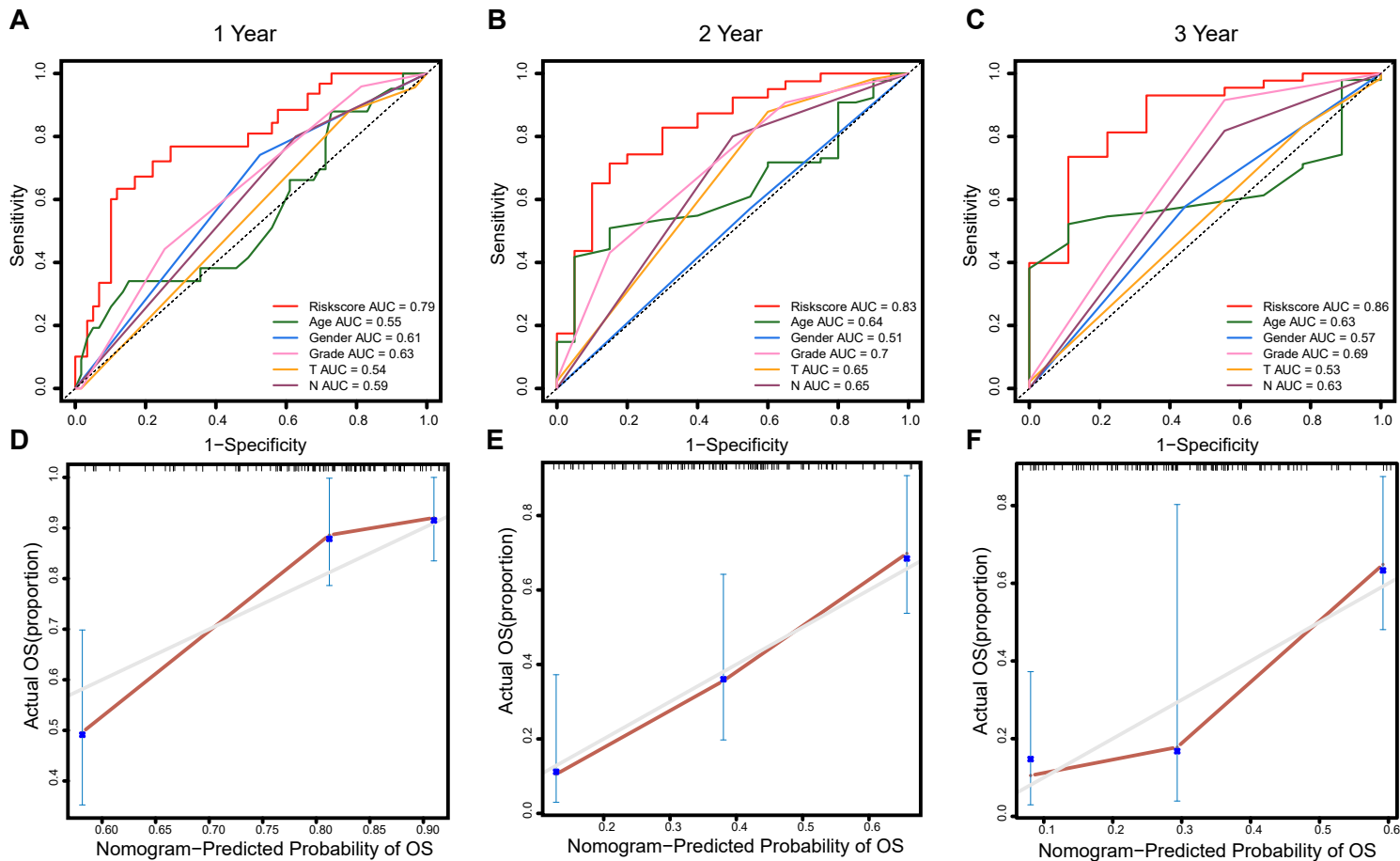

Supplement: Supplementary Materials — Supplementary Material S1: functional analysis of 710 differentially expressed mRNA (DEms) between low-risk and high-risk subgroups. Supplementary Material S2: EMT-LPS was an independent prognostic factor for PAAD patients. Supplementary Material S3: construction of EMT-LPS calibration in TCGA dataset. Supplementary Material S4: nomogram and calibration based on EMT-LPS in the ICGC dataset. Supplementary Material S5: (A, B) Principal component analysis of EMT-LPS in TCGA and ICGC datasets. (C) Heatmap of the relationship between the expression levels of eleven EMT-related lncRNAs and clinicopathological features in the ICGC dataset. (D-G) Stratification analysis of EMT-LPS in ICGC patients with different clinical characteristics. [file 8259951.f1.zip › Supplementary material S3.pdf]

Supplementary material S4

A

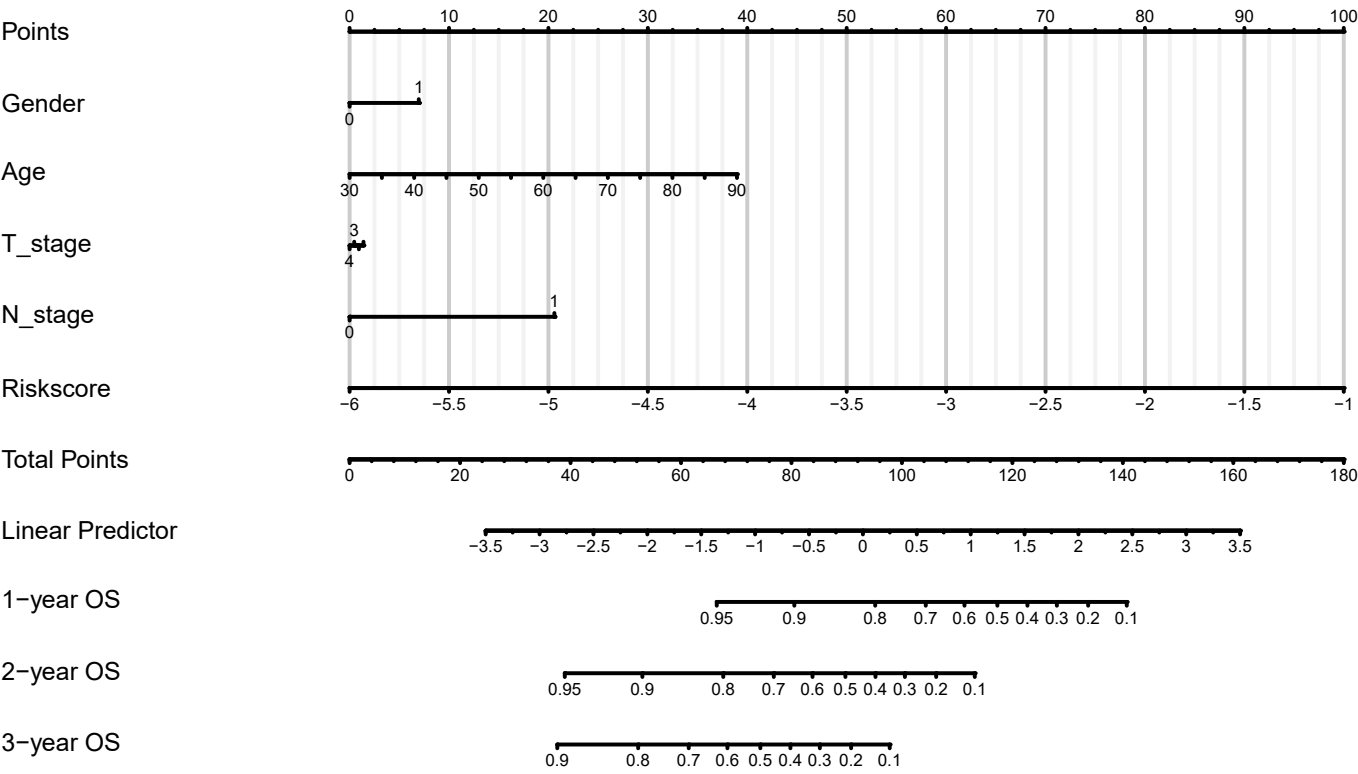

B

1 Year

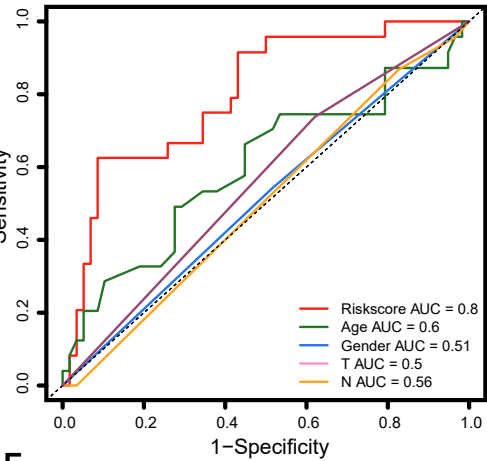

C

2 Year

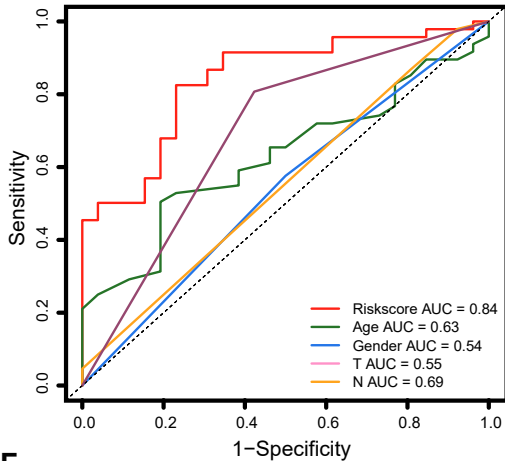

D

3 Year

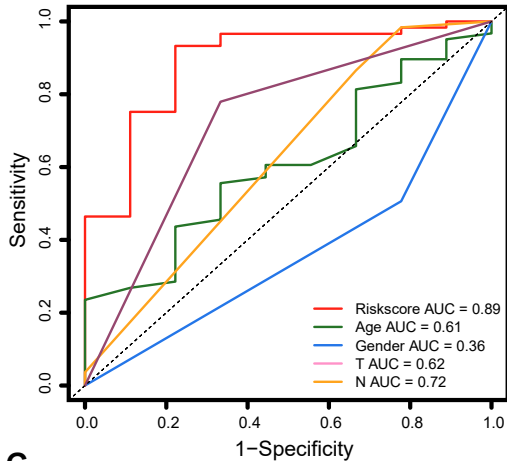

E

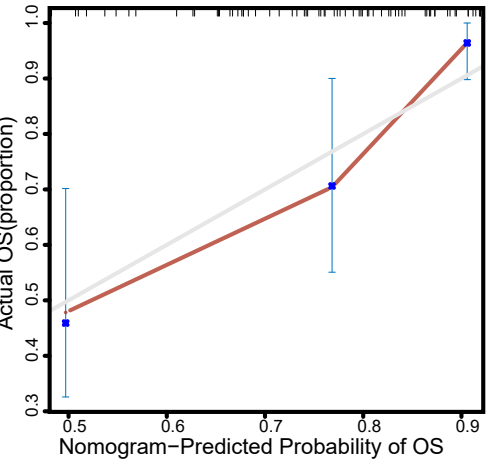

F

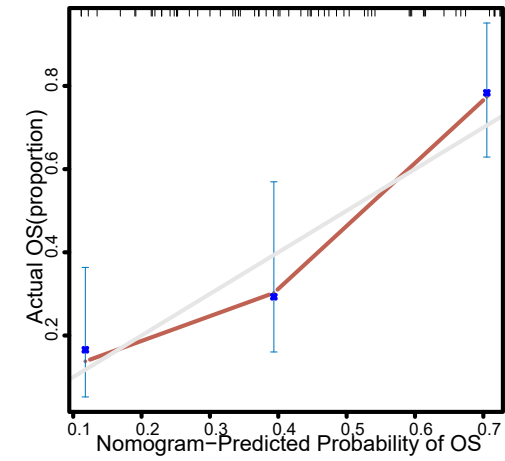

G

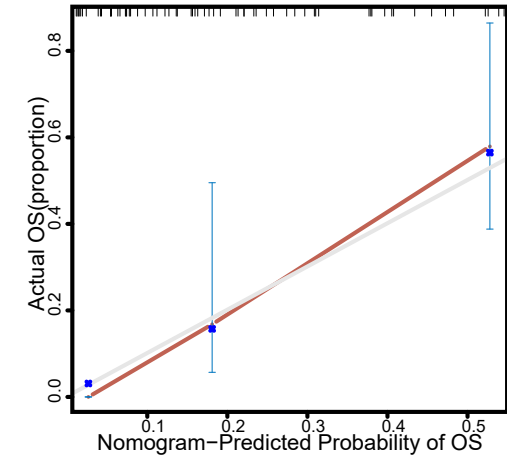

Supplement: Supplementary Materials — Supplementary Material S1: functional analysis of 710 differentially expressed mRNA (DEms) between low-risk and high-risk subgroups. Supplementary Material S2: EMT-LPS was an independent prognostic factor for PAAD patients. Supplementary Material S3: construction of EMT-LPS calibration in TCGA dataset. Supplementary Material S4: nomogram and calibration based on EMT-LPS in the ICGC dataset. Supplementary Material S5: (A, B) Principal component analysis of EMT-LPS in TCGA and ICGC datasets. (C) Heatmap of the relationship between the expression levels of eleven EMT-related lncRNAs and clinicopathological features in the ICGC dataset. (D-G) Stratification analysis of EMT-LPS in ICGC patients with different clinical characteristics. [file 8259951.f1.zip › Supplementary material S4.pdf]

# Supplementary material S5

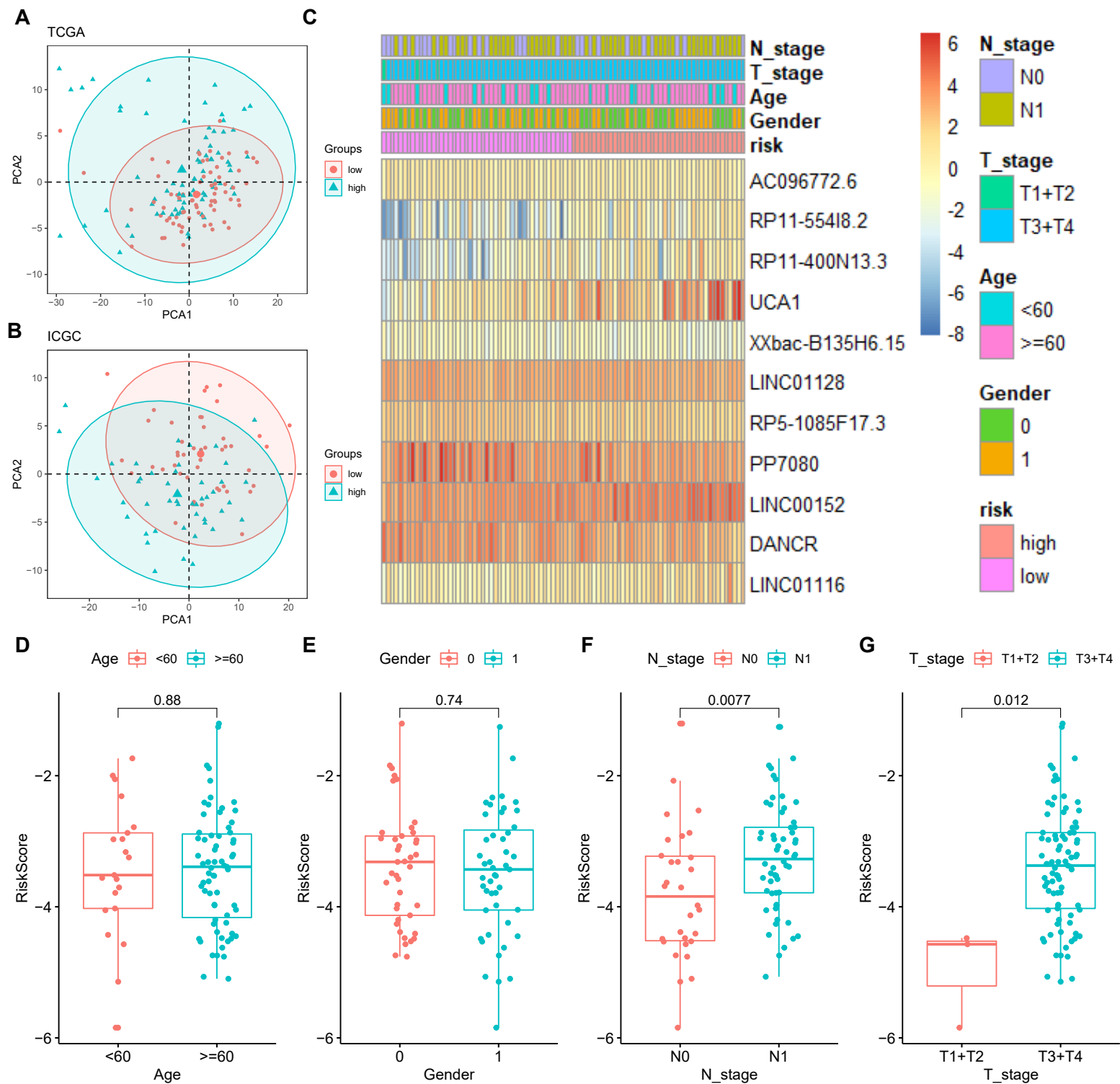

Supplement: Supplementary Materials — Supplementary Material S1: functional analysis of 710 differentially expressed mRNA (DEms) between low-risk and high-risk subgroups. Supplementary Material S2: EMT-LPS was an independent prognostic factor for PAAD patients. Supplementary Material S3: construction of EMT-LPS calibration in TCGA dataset. Supplementary Material S4: nomogram and calibration based on EMT-LPS in the ICGC dataset. Supplementary Material S5: (A, B) Principal component analysis of EMT-LPS in TCGA and ICGC datasets. (C) Heatmap of the relationship between the expression levels of eleven EMT-related lncRNAs and clinicopathological features in the ICGC dataset. (D-G) Stratification analysis of EMT-LPS in ICGC patients with different clinical characteristics. [file 8259951.f1.zip › Supplementary material S5.pdf]
